# Genetic Structure of *Eudorella emarginata* (Peracarida: Cumacea): Effects of Topography and Historical Anoxic Events in the Sea of Japan

**DOI:** 10.1002/ece3.72098

**Published:** 2025-09-08

**Authors:** Kai Okamoto, Shigeaki Kojima

**Affiliations:** ^1^ Atmosphere and Ocean Research Institute The University of Tokyo Kashiwa Chiba Japan

**Keywords:** anoxia, biogeography, Cumacea, deep sea, refugium, Sea of Japan

## Abstract

Geohistorical events are among the most important factors determining population genetic structure. The Sea of Japan is an intriguing area because of its connection to neighboring seas via shallow straits (< 140 m deep) and the occurrence of deep‐water anoxic events during glacial periods. Despite repeated anoxic events, species with low dispersal capabilities have been reported at depths deeper than the straits in both the Sea of Japan and the Pacific Ocean. We focused on one such species, *Eudorella emarginata* (Cumacea, Peracarida). This species is expected to have limited dispersal capabilities owing to its lack of a planktonic larval stage. Phylogenetic analyses based on mitochondrial and nuclear DNAs revealed three distinct clades within individuals of *E. emarginata* distributed in Japanese waters, suggesting the existence of sibling species. Only one clade included individuals from both the Sea of Japan (519–1024 m deep) and the Pacific Ocean (490–1504 m deep). Further analyses focused on this clade to explore the relationship between population dynamics and geohistorical events. The effects of glacial anoxia have been suggested in Sea of Japan populations; however, such an effect has not been detected in a population nearby the Tsugaru Strait. We hypothesized that the inflow of oxygen‐rich seawater from the Tsugaru Strait maintained suitable conditions in the area facing the strait during glacial periods. Future studies should implement genome‐wide strategies to investigate this hypothesis by focusing on high‐resolution genetic structure.

## Graphical abstract

The population dynamics of cumacean species *Eudorella emarginata* in the seas around Japan were estimated based on molecular sequence data and past climatic changes. Our results suggest that the population in the Sea of Japan survived glacial anoxic events in the refugium near the Tsugaru strait. Our results emphasize the importance of local populations near the strait for considering population dynamics of deep‐sea benthic organisms in the Sea of Japan.

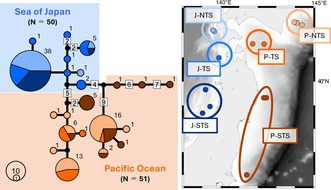

## 1 Introduction

Historical fluctuations in populations were recorded as geographical genetic structures. Comparison of these structures with geohistorical events enabled us to reconstruct the formation process of the current distribution. Consistent trends are often observed across multiple taxa, despite interspecific differences in structure, such as latitudinal and altitudinal range shifts related to the glacial cycle (Hewitt 2004). Thus, the accumulation of detailed information on many species could contribute to the recognition of broad patterns across taxa. From a biogeographical perspective, the Sea of Japan has a significant historical background regarding its formation and environmental changes. These features provide valuable insights into the relationship between population dynamics and historical events.

**FIGURE 1:**
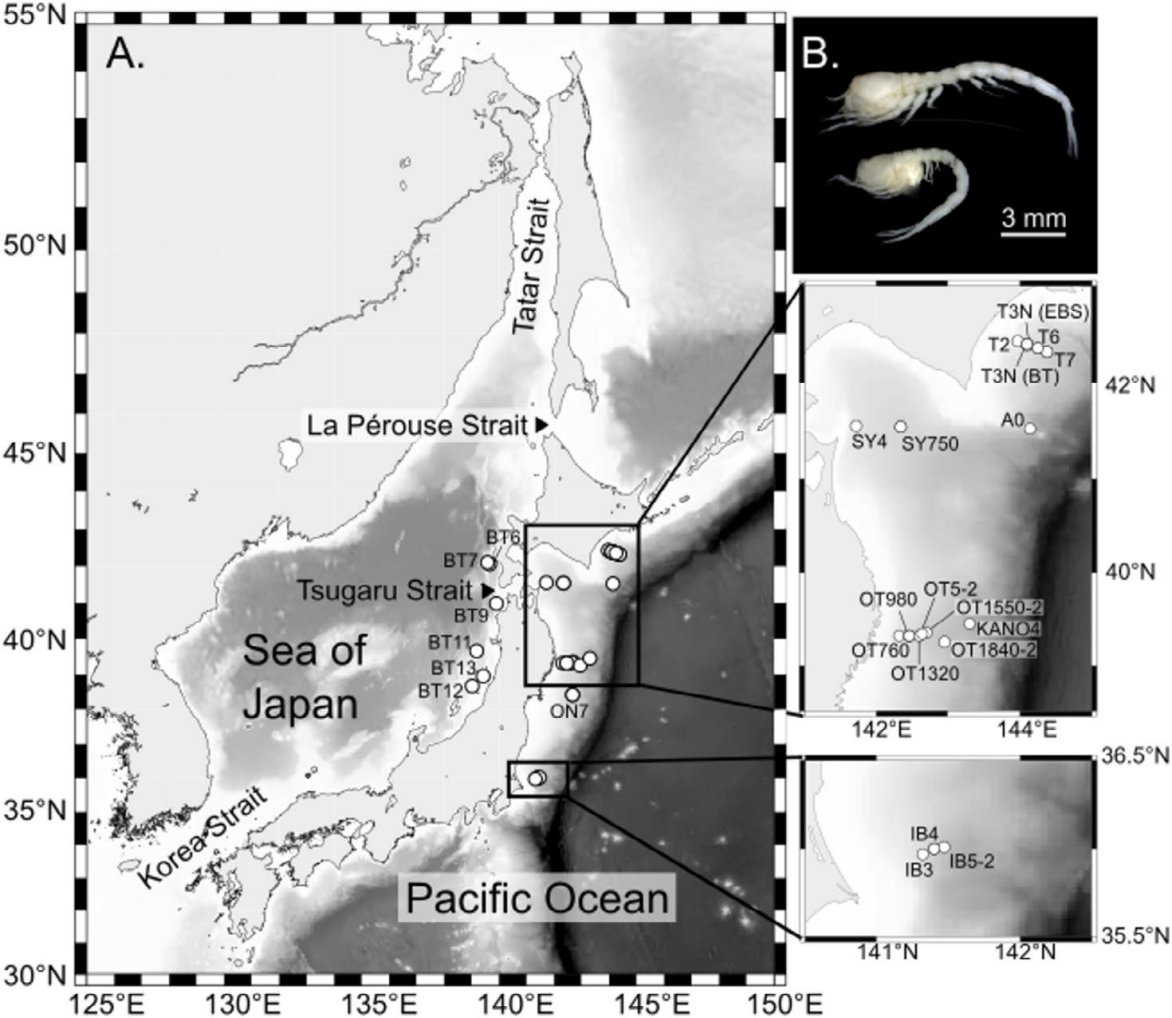
Sampling sites (A) and “ *Eudorella emarginata* ” (B). Specimens were collected from 25 stations: Six stations in the Sea of Japan and 19 stations in the Pacific Ocean. The map was created using GMT 6 (Wessel et al. 2019) based on bathymetry data from ETOPO1 (Amante and Eakins 2009). For further information, see Table 1.

The Sea of Japan (Figure 1A) was transformed into a semi‐enclosed marginal sea by the uplift of the northern part of the Japanese Archipelago approximately 4.5–1.7 million years ago (Sato et al. 2012). The deep waters (deeper than 200 m) of the Sea of Japan remain isolated from the Pacific Ocean despite sea level fluctuations during the glacial–interglacial cycles (Oba and Irino 2012; Miller et al. 2020). The Sea of Japan is currently connected to adjacent seas through several shallow and narrow straits, including the Tsugaru Strait (~130 m deep), Korea Strait (~130 m deep), Kanmon Straits (~50 m deep), La Pérouse Strait (~50 m deep), and Tatar Strait (~10 m deep). This unique topography may act as a barrier to the dispersal of marine species, particularly those inhabiting deeper waters throughout their lifecycle. Thus, the Sea of Japan is a suitable region for studying the processes and mechanisms of speciation due to its geographical isolation in the deep sea.

Another notable feature of the Sea of Japan is the occurrence of multiple glacial deep‐water anoxic events (Tada 1994; Oba and Irino 2012; Itaki 2016). An anoxic event that occurred during the last glacial period is a well‐studied example of this phenomenon. The sea level dropped by up to 120 ± 7 m at the last glacial maximum (LGM) (Oba and Irino 2012), limiting the exchange of seawater between the Sea of Japan and the Pacific Ocean. At the same time, freshwater flowed into the Sea of Japan from the Asian continent and created a stratified structure comprising a surface freshwater layer and an underlying seawater layer. Consequently, the bottom layer of the Sea of Japan became anoxic because the structure inhibited vertical mixing of seawater (Itaki et al. 2004). These anoxic episodes have affected the survival of various marine animals and, in some cases, may have led to local extinction (Amano 2004).

Many studies have suggested that oxygen‐rich seawater may have existed in the interior of the Sea of Japan and/or nearby waters during glacial periods (Gorbarenko and Southon 2000; Itaki et al. 2004). These areas served as refugia, allowing certain species to survive. Several studies have suggested the existence of refugia in the southern and/or northern regions of the Sea of Japan (e.g., Hirase and Ikeda 2014; Hirase et al. 2016; Fujita et al. 2021). The southern refugium is thought to have been formed by constant current inflow via the Korea Strait (Kojima et al. 2001; Hirase and Ikeda 2014). Hirase et al. (2016) examined genetically distinct populations of the shallow‐water goby, *Chaenogobius annularis* , in both the northern and southern regions and suggested the existence of another refugium in the northern part of the Sea of Japan. This northern refugium is supported by two deep‐sea crangonid shrimps of the genus *Argis* (Fujita et al. 2021). However, these studies did not consider the factors that formed the northern refugium. Some studies indicated that species distributed in a specific depth range tend to survive during glacial periods and hypothesized that an exceptionally oxic seawater layer existed between the surface freshwater layer and anoxic bottom water at a depth of approximately 100–400 m (Amano 2004). Fujita et al. (2021) reported that the impact of anoxia was smaller for shallow‐distributed species (200–300 m) than for deep‐distributed species (250–2000 m).

Most studies on refugia in the Sea of Japan have focused on species distributed at depths shallower than or similar to those of the straits (Kokita and Nohara 2011; Hirase et al. 2016; Sakuma et al. 2019) or species with a planktonic larval stage (Fujita et al. 2017, 2021). Other studies have also lacked comparisons with the same species or sister groups from the Pacific Ocean (Amano 2004; Iguchi et al. 2007). Therefore, the possibility of post‐LGM colonization could not be excluded.

Peracarids represent the principal components of deep‐sea benthic ecosystems (Brandt et al. 2019). They are characterized by a unique reproductive strategy that utilizes a brood pouch (marsupium) for parenting. Juveniles grow inside the pouch and hatch as miniature adults called manca juveniles. Because of their reproductive strategy, benthic peracarids are expected to have low dispersal capabilities (Sanders and Grassle 1971). *Eudorella emarginata* (Figure 1B) is a benthic peracarid species distributed in the mid‐to‐high latitudes of the Northern Hemisphere, including the Sea of Japan and the northwestern Pacific Ocean (Watling and Gerken 2025). The generation time of cumaceans generally ranges from several months to 1 year; however, some deep‐sea species can live for three or more years (Bishop and Shalla 1994). Similar to other peracarids, *E. emarginata* lacks planktonic larval stages and completes its entire life cycle in a benthic environment. This is an interesting subject for phylogeographic studies. Around Japan, this species is found at depths greater than that of the straits (Sea of Japan: 383–1564 m, Pacific Ocean: 965–2183 m) (Akiyama 2009, 2014; Akiyama and Gamô 2012), enabling a phylogeographic comparison between conspecific populations in the Sea of Japan and the Pacific Ocean. Akiyama and Gamô (2012) noted that the *E. emarginata* from the Pacific Ocean is morphologically different from that in the original description, the type specimen collected in the Øresund Strait in the North Sea (Krøyer 1846). Multiple intraspecific genetically divergent lineages and/or cryptic species may exist within the morphological species “ *E. emarginata* .”

As a first step in phylogeographic studies of this species, we analyzed the genetic structure of *“E. emarginata* ” individuals inhabiting Japanese waters. Based on the phylogeny, a biogeographic analysis focusing on lineages containing individuals from the Sea of Japan was conducted to investigate the population dynamics and effects of anoxic events in the Sea of Japan. We hypothesized that individuals inhabiting the Sea of Japan, geographically isolated by the Japanese archipelago, exhibit significant genetic divergence from individuals in other sea areas and that they survived anoxic conditions during glacial periods. Furthermore, given that the Korea Strait is known to contribute to the survival of other species in glacial anoxia, we predicted that the Tsugaru Strait would serve as a comparable refugium for the Sea of Japan population.

## 2 Materials and Methods

### 2.1 Sample Collection

Specimens were collected around the northeastern part of Japan, including the Sea of Japan and the Pacific Ocean. Specimens were collected during deep‐sea expeditions KS‐15‐10, KS‐18‐8, KS‐19‐7, KS‐19‐20, KS‐20‐15, KS‐20‐18, KS‐21‐13, KS‐21‐14, and KH‐22‐8 (Figure 1, Table 1). Sampling was conducted using a 3 m beam trawl and plankton nets with a mesh size of 0.33–0.5 mm attached to the trawl (Akiyama et al. 2008). During the KH‐22‐8 expedition, a 4 m beam trawl was used instead of a 3 m beam trawl. An epibenthic sledge (Brenke 2005) with 0.5 mm mesh size nets was also used in the KS‐20‐18 expedition for sampling. The specimens were fixed in 70% or 99.5% ethanol on board, sorted, and refixed in 99.5% ethanol in the laboratory at the Atmosphere and Ocean Research Institute, the University of Tokyo.

**TABLE 1:** List of sampling sites. The “Number of specimens of each clade” columns refer to sequenced individuals, while “local population” indicates the subset of Clade 1 specimens analyzed in the haplotype network and neutrality tests. In the Gear column, BT and EBS indicate beam trawl and epibenthic sledge, respectively.

Expedition	Station	Region	Local population	Number of specimen of each clade			Coordinate and depth on bottom			Coordinate and depth off bottom			Gear
				Clade 1	Clade 2	Clade 3	Latitude	Longitude	Depth (m)	Latitude	Longitude	Depth (m)	
KS‐15‐10	ON7	Pacific Ocean	—	0	3	1	38°25.38′ N	142°40.40′ E	1369	38°25.69′ N	142°39.55′ E	1345	3 m BT
KS‐18‐8	OT5‐2	Pacific Ocean	—	0	2	0	39°20.52′ N	142°38.68′ E	1458	39°19.51′ N	142°38.33′ E	1424	3 m BT
KS‐18‐8	KANO4	Pacific Ocean	—	0	2	0	39°27.12′ N	143°17.70′ E	2348	39°27.27′ N	143°16.44′ E	2301	3 m BT
KS‐19‐7	OT980	Pacific Ocean	P‐STS	3	7	2	39°19.03′ N	142°27.70′ E	997	39°20.26′ N	142°27.91′ E	1005	3 m BT
KS‐19‐20	SY4	Pacific Ocean	P‐TS	1	2	0	41°32.43′ N	142°20.11′ E	1198	41°33.33′ N	142°20.01′ E	1194	3 m BT
KS‐19‐20	SY750	Pacific Ocean	P‐TS	10	0	0	41°32.81′ N	141°43.28′ E	749	41°33.69′ N	141°43.48′ E	753	3 m BT
KS‐20‐15	OT760	Pacific Ocean	P‐STS	1	0	0	39°18.98′ N	142°19.34′ E	761	39°19.81′ N	142°19.54′ E	765	3 m BT
KS‐20‐15	OT1320	Pacific Ocean	—	0	1	0	39°19.49′ N	142°35.71′ E	1337	39°20.81′ N	142°35.76′ E	1306	3 m BT
KS‐20‐15	OT1550‐2	Pacific Ocean	—	0	1	0	39°20.97′ N	142°42.05′ E	1559	39°19.96′ N	142°41.73′ E	1557	3 m BT
KS‐20‐15	OT1840‐1	Pacific Ocean	—	0	3	0	39°17.50′ N	142°56.88′ E	1850	39°16.36′ N	142°56.79′ E	1831	3 m BT
KS‐20‐18	T2	Pacific Ocean	P‐NTS	1	0	0	42°26.07′ N	143°58.32′ E	504	42°26.83′ N	143°58.68′ E	490	3 m BT
KS‐20‐18	T3NBT	Pacific Ocean	P‐NTS	12	4	0	42°23.58′ N	144°05.71′ E	847	42°24.38′ N	144°06.25′ E	827	3 m BT
KS‐20‐18	T3N	Pacific Ocean	P‐NTS	12	1	1	42°24.05′ N	144°05.92′ E	826	42°24.91′ N	144°06.46′ E	823	EBS
KS‐20‐18	T6	Pacific Ocean		0	5	1	42°21.57′ N	144°14.73′ E	1115	42°22.43′ N	144°15.48′ E	1161	3 m BT
KS‐20‐18	T7	Pacific Ocean	P‐NTS	1	1	0	42°19.30′ N	144°22.96′ E	1402	42°20.47′ N	144°23.58′ E	1412	3 m BT
KS‐21‐13	BT6	Sea of Japan	J‐NTS	10	0	0	42°03.53′ N	139°39.88′ E	757	42°02.81′ N	139°39.71′ E	765	3 m BT
KS‐21‐13	BT7	Sea of Japan	J‐NTS	10	0	0	42°05.83′ N	139°34.99′ E	599	42°05.39′ N	139°34.99′ E	587	3 m BT
KS‐21‐13	BT9	Sea of Japan	J‐TS	15	0	0	40°58.81′ N	139°53.95′ E	801	40°59.23′ N	139°54.05′ E	788	3 m BT
KS‐21‐13	BT11	Sea of Japan	J‐STS	4	0	0	39°39.35′ N	139°11.00′ E	1025	39°39.13′ N	139°11.38′ E	1022	3 m BT
KS‐21‐13	BT12	Sea of Japan	J‐STS	10	0	0	38°56.97′ N	139°25.07′ E	519	38°57.28′ N	139°25.29′ E	528	3 m BT
KS‐21‐13	BT13	Sea of Japan	J‐STS	1	0	0	38°40.39′ N	139°01.22′ E	563	38°39.87′ N	139°00.06′ E	570	3 m BT
KS‐21‐14	IB3	Pacific Ocean	—	0	0	1	35°58.37′ N	141°19.04′ E	1066	35°58.87′ N	141°19.04′ E	1064	3 m BT
KS‐21‐14	IB4	Pacific Ocean	P‐NTS	10	10	0	36°00.10′ N	141°24.07′ E	1504	35°59.73′ N	141°24.03′ E	1499	3 m BT
KS‐21‐14	IB5‐2	Pacific Ocean	—	0	1	0	36°00.44′ N	141°27.92′ E	1816	35°59.70′ N	141°27.92′ E	1822	3 m BT
KH‐22‐8	A0	Pacific Ocean	—	0	1	0	41°31.36′ N	144°08.60′ E	1987	41°30.97′ N	144°08.17′ E	2007	4 m BT

Species identification was conducted based on the identification key and description provided in the report on *Eudorella* species from Japanese waters (Akiyama and Gamô 2012). *E. emarginata* is characterized by a non‐serrated frontal margin with a large sinus in females and is distinguished from other similar species by the accessory flagellum of the antenna 1 being shorter than the first article of the main flagellum. While identification relied on mature female individuals, immature and male specimens were included in the molecular phylogenetic analyses to increase the sample size.

### 2.2 DNA Extraction, PCR Amplification and Sequencing

“ *Eudorella emarginata* ” is distributed across multiple oceanic regions, and morphological differences between regions have been noted (Akiyama and Gamô 2012). In the present study, we reconstructed the phylogeny of “ *E. emarginata* ” based on the nucleotide sequences of mitochondrial and nuclear DNAs. Phylogeographic analyses were conducted, focusing on a clade that included individuals from the Sea of Japan. We selected at least two individuals from each site for DNA extraction.

Total DNA was extracted from pereiopod or pleonite tissue using a DNeasy Blood and Tissue Kit (Qiagen, Hilden, Germany). The DNA extracts were stored at −20°C. A partial DNA fragment of the mitochondrial cytochrome c oxidase subunit I (COI) region was amplified by using primer set LCO1490 (5′‐GGT CAA CAA ATC ATA AAG ATA TTG G‐3′) (Folmer et al. 1994) and HCO2198 (5′‐TAA ACT TCA GGG TGA CCA AAA AAT CA‐3′) (Folmer et al. 1994). Partial DNA fragments of the nuclear 28S rDNA (28S) and internal transcribed spacer 2 rDNA (ITS2) were amplified for each monophyletic group identified by analysis of COI by using primer set 1274 (5′‐GAC CCG TCT TGA AAC ACG GA‐3′) (Whiting et al. 1997) and FF (5′‐GGT GAG TTG TTA CAC ACT CCT TAG TCG GAT‐3′) (Jarman et al. 2000) and primer sets ITSEe (5′‐CGG TAA GAT TCT TGT ATC TGA GC‐3′) (originally designed for *E. emarginata* ) and ITS4 (5′‐TCC TCC GCT TAT TGA TAT GCT‐3′) (White et al. 1990), respectively.

Polymerase chain reactions were performed in 25 μL reaction volumes containing 2.5 μL DNA extract, 2.5 μL PCR buffer, 2.0 μL dNTP mixture, 0.3 μL forward and reverse primers (20 μM each) and 0.1 μL Ex Taq Hot Start Version (TaKaRa Bio, Shiga, Japan). The cycling parameters were as follows: initial denaturation at 94°C for 120 s, followed by 35 cycles consisting of denaturation at 98°C for 30 s, annealing at 42°C (COI) or 50°C (28S and ITS2) for 40 s, and extension at 72°C for 60 s.

PCR products were purified using Exo‐sap Express (Thermo Fisher Scientific, Waltham, MA, USA) according to the manufacturer's protocol. Sequencing was performed using the BigDye Terminator Cycle Sequence Kit v3.1 (Thermo Fisher Scientific) with the same primers as those used for PCR. The products were purified using the BigDye Xterminator Purification Kit (Thermo Fisher Scientific). Nucleotide sequences were determined using the ABI 3130xl and ABI 3500 sequencers at the Atmosphere and Ocean Research Institute. GenBank sequence data of the individuals of *E. emarginata* from the northern Atlantic Ocean were included in the phylogenetic analysis (Table 2). All sequences were aligned based on the ClustalW algorithm implemented in MEGA 11 version 11.0.11 (Tamura et al. 2021). Following sequence alignment, only datasets with indels were trimmed using Gblocks version 0.91b (https://www.biologiaevolutiva.org/jcastresana/Gblocks.html, accessed July 6, 2025) with the default parameters for subsequent analysis. All sequence data were submitted to the GenBank database under the accession numbers listed in Table S1.

**TABLE 2:** List of GenBank accession numbers included in this study. Sequences newly generated for this study are listed in Table S1.

Species	Location of collection	COI	28S
*Eudorella emarginata*	Fensfjorden, Norway	MK757516	MK644846
*Eudorella emarginata*	Skagerak, Norway	MK757513	MK644851
*Eudorella emarginata*	Skagerak, Norway	MK757515	MK644852
*Eudorella emarginata*	Svalbard, Norway	MK757514	—
*Eudorella emarginata*	Kattegatt, Sweden	MG935157	—
*Eudorella hirsuta*	Skagerak, Norway	MK757543	—
*Eudorella hirsuta*	Fensfjorden, Norway	MK757544	—
*Eudorella pusilla*	Walpole, ME, USA	AF137516	—

### 2.3 Phylogenetic Analysis

Two maximum likelihood trees were constructed using raxml GUI v2.0.10 (Edler et al. 2021): one based on the COI sequences (617 bp) and another based on the combined 28S (331 bp) and ITS2 (367 sites) sequences. The sequences of the genus *Eudorella* from GenBank were used as outgroups (Table 2). The best fitting substitution model was identified using ModelTest‐NG v0.1.7 based on the Akaike information criterion. The general time reversible model with a gamma distribution and the Hasegawa‐Kishino‐Yano model with a gamma distribution were identified as the best‐fitting substitution models, respectively. These phylogenetic trees were visualized using FigTree v1.4.4 (http://tree.bio.ed.ac.uk/software/figtree/, accessed July 6, 2025).

Based on COI sequences, a TCS haplotype network, generated with Popart ver. 1.7 (Leigh and Bryant 2015), was used to visualize the relationships among haplotypes within the monophyletic group (including individuals from the Sea of Japan).

Divergence times were estimated using IMa3 (Hey et al. 2018) with a divergence rate of 5.0%–5.2%/Ma, estimated for Arctic crustaceans around the Bering Strait (Loeza‐Quintana et al. 2019). Given its circumboreal distribution, this calibration was judged more appropriate for “ *E. emarginata* ” than the conventionally applied rate for marine taxa, 1.4%/Ma (Knowlton and Weigt 1998). Initial IMa3 analyses employed a uniform prior for descendant population sizes (*θ* ~ *U* [0, 50]), migration rates (*m* ~ *U* [0, 0.5]), and divergence time (*t* ~ *U* [0, 40]). Whenever a parameter's posterior distribution abutted its upper bound, its prior limit was doubled and the analysis was repeated. The heating scheme was configured according to the IMa3 manual. We discarded the first 10% of MCMC samples as burn‐in, logged parameter estimates every 10,000 steps, and sampled genealogies every 100 steps. Convergence was confirmed by ensuring effective sample sizes (ESS) exceeded 200 for all key parameters. We grouped the sampling sites into three local populations in the Sea of Japan (J) and Pacific Ocean (P): those near the Tsugaru Strait (J‐TS and P‐TS), areas north of the Tsugaru Strait (J‐NTS and P‐NST), and areas south of the strait (J‐STS and P‐STS). Pairwise *F*_ST_ values among the six populations were calculated using Arlequin v3.5.2.2 (Excoffier and Lischer 2010) using corrected Kimura's two‐parameter distances and *p*‐values were Bonferroni corrected for multiple comparisons. For each population, we estimated the haplotype diversity (Hd), nucleotide diversity (Pi), Tajima's *D*, Fu's *F*_s_, and Fu and Li's *F* using DnaSP ver. 6.12 (Rozas et al. 2017).

## 3 Results

**FIGURE 2:**
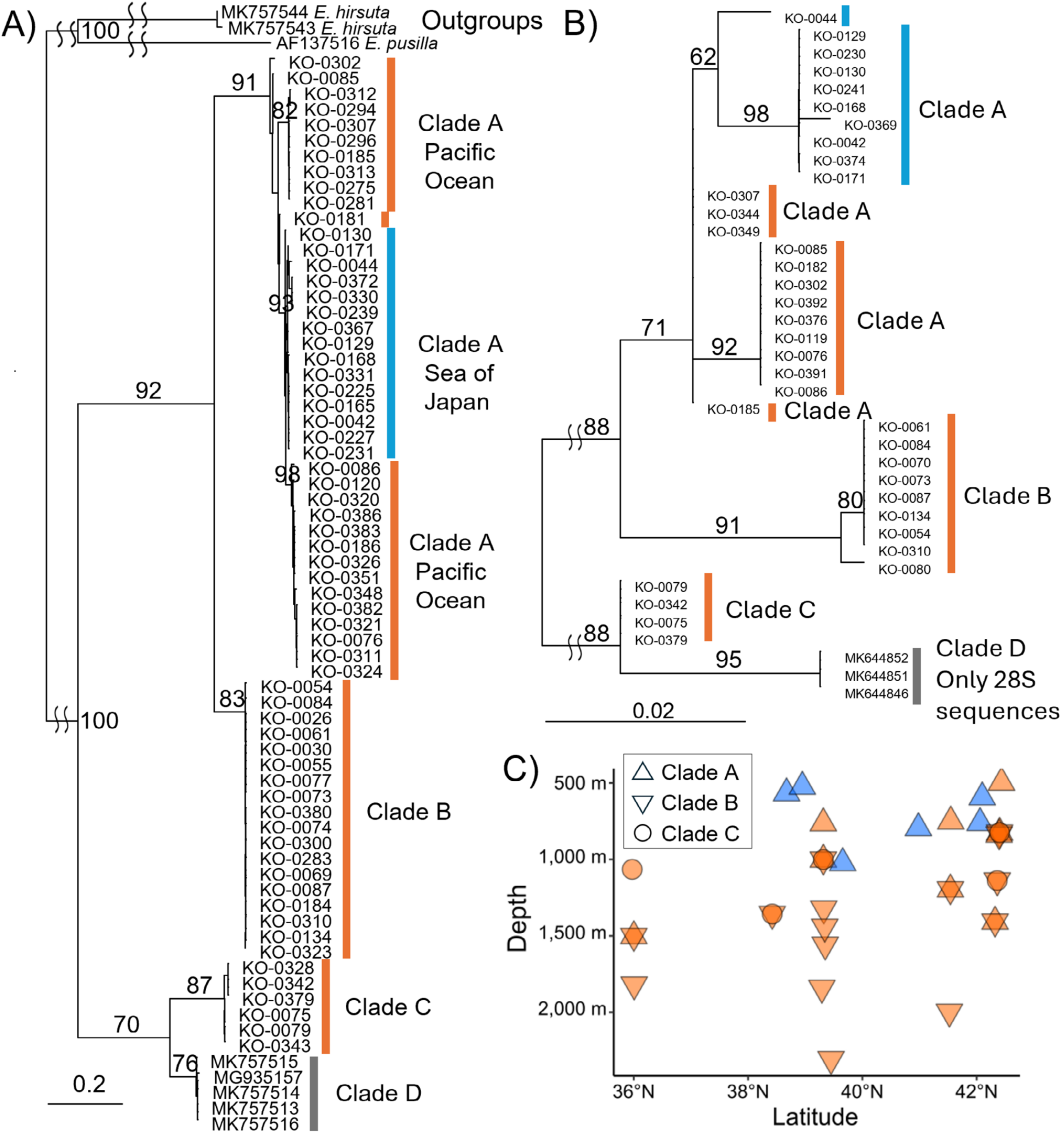
The phylogenetic relationship in the “ *Eudorella emarginata* ” and collected sites of each clade. Colors indicate sampling sea areas; blue, orange, and gray indicate the Sea of Japan, Pacific Ocean, and Atlantic Ocean, respectively. (A) ML tree based on the mitochondrial DNA (COI: 617 bp). When multiple individuals with a perfectly matching haplotype were obtained from a single sampling site, the phylogenetic tree was constructed using only one individual. (B) ML tree based on the nuclear DNA (28S: 331 bp; ITS2: 367 sites). The branch values are indicative of the respective bootstrap values for major clades. (C) Graph displaying the latitudinal bathymetrical distributions for each Japanese clades, indicated by distinct symbols (△, ▽, and ○).

**FIGURE 3:**
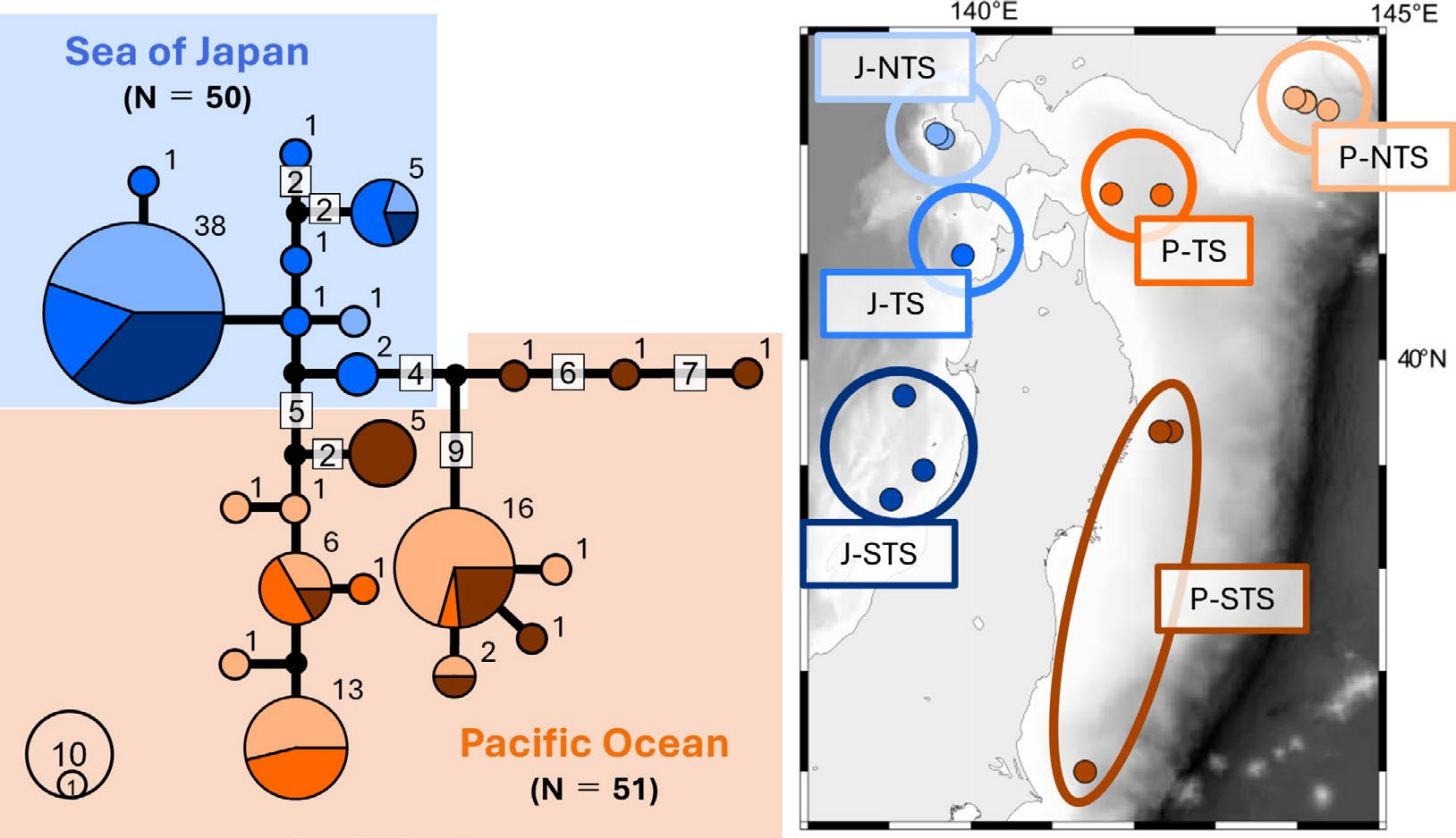
TCS haplotype network based on the mitochondrial DNA (COI: 658 bp), focusing on Clade A. Each circle represents a specific haplotype. The circle size and number at the top right corner of each circle refers to the number of individuals sharing that haplotype. If two or more nucleotides differ between haplotypes, the number of different bases is indicated by a number in the square. The colors of sectors correspond to the location of sampling sites as shown in the map. The black circles in the network represent undiscovered haplotypes.

The present molecular analysis showed that “ *E. emarginata* ” inhabiting Japanese waters consists of three clades (Figure 2A), namely Clade A, which is distributed in both the Pacific Ocean and the Sea of Japan (490–1504 and 519–1024 m, respectively), Clade B distributed in deeper depths of the Pacific Ocean (822–2348 m), and Clade C distributed in shallower depths of the Pacific Ocean (822–1357 m). These clades were also supported by nuclear DNAs (Figure 2B). The maximum depth recorded in Clade B updates the bathymetric distribution of “ *E. emarginata* ” in the Northwest Pacific (2183 m; Akiyama 2009). The bathymetric distribution ranges of these three Japanese clades overlapped, and individuals from more than one clade were sampled at single sampling sites (Figure 2C). “ *E. emarginata* ” from northern Atlantic waters formed a monophyletic group (Clade D), to which the northwestern Pacific clade (Clade C) was more closely related than the other two Japanese clades. Some COI haplotypes of Clade A were obtained from the northernmost to southernmost sites within the Pacific Ocean and Sea of Japan, whereas no COI haplotypes were shared between these two sea area populations (Figures 2 and 3). These trends were observed in the nuclear DNAs datasets (Figure 2).

Divergence ages were estimated for the two evolutionary splits. The initial divergence between Clades A and B was estimated to be ~2.72 Ma, a time that aligns with the isolation event of the Sea of Japan. A secondary split within Clade A between populations in the Sea of Japan and Pacific Ocean was estimated to be ~0.42 Ma.

Pairwise *F*_ST_ analyses revealed strong and significant differentiation between the two major regions (Sea of Japan vs. Pacific), whereas comparisons within each region showed low, non‐significant *F*_ST_ values (Table 3). Local populations in the Pacific Ocean showed high haplotype diversity (Table 4). P‐NTS showed significantly positive values for both Tajima's *D* and Fu's *F*_s_, whereas P‐TS exhibited significantly negative values for both Tajima's *D* and Fu and Li's *F* (Table 4). Local populations in the Sea of Japan exhibited low haplotype diversity and significantly negative values for both Tajima's *D* and Fu and Li's *F* (Table 4), except for the local population J‐TS, which showed a high haplotype diversity comparable to those of the local populations in the Pacific Ocean and positive values for both Tajima's *D* and Fu and Li's *F* (Table 4).

**TABLE 3:** Pairwise *F*_ST_ values among local populations within Clade A.

	J‐NTS	J‐TS	J‐STS	P‐NTS	P‐TS	P‐STS
J‐NTS	—					
J‐TS	0.157	—				
J‐STS	−0.051	0.141	—			
P‐NTS	0.481*	0.363*	0.443*	—		
P‐TS	0.781*	0.659*	0.764*	0.313	—	
P‐STS	0.494*	0.402*	0.466*	0.275	0.033	—

* Bonferroni‐corrected *p* < 0.05.

**TABLE 4:** Haplotype diversity, nucleotide diversity, Tajima's *D*, Fu's *F*_s_, and Fu and Li's *F* of each local population.

Station	*N*	nHP	Hd	Pi	Tajima's *D*	*p*	Fu's *F*_s_	*p*	Fu and Li's *F*	*p*
Overall	101	22	0.814	0.015	0.180	NS	1.264	NS	−0.473	NS
Pacific Ocean	51	14	0.824	0.019	0.967	NS	4.611	< 0.05	0.115	NS
P‐NTS	26	8	0.729	0.018	2.558	< 0.01	6.315	< 0.01	1.559	NS
P‐TS	11	4	0.673	0.008	−1.735	< 0.05	3.395	NS	−2.415	< 0.05
P‐STS	14	8	0.857	0.021	0.730	NS	2.553	NS	0.395	NS
Sea of Japan	50	8	0.417	0.002	−1.139	NS	−1.451	NS	−1.122	NS
J‐NTS	20	4	0.284	0.001	−1.972	< 0.05	−0.545	NS	−2.680	< 0.05
J‐TS	15	6	0.762	0.004	0.147	NS	0.693	NS	0.425	NS
J‐STS	15	2	0.133	0.001	−1.911	< 0.05	1.738	NS	−2.671	< 0.05

Abbreviations: Hd, haplotype diversity; *N*, sample size; nHP, number of haplotypes; NS, not significant; Pi, nucleotide diversity.

## 4 Discussion

Widely distributed species often contain several cryptic species (Brandt et al. 2012). Despite the analysis focusing on only a part of the wide distribution area of “ *E. emarginata* ” we found three monophyletic clades that differed by more than 9% in the nucleotide sequences of the mitochondrial COI region (Figure 2A). Each monophyletic clade could also be distinguished from the other clades based on the nuclear 28S and ITS2 ribosomal DNAs (Figure 2B). As mature individuals of different clades coexisted (Figure 2C), the coincidence of genetic deviations between mitochondrial and nuclear DNAs indicated that they were reproductively isolated from each other. Based on the concept of biological species, it may be valid to consider these three clades to be different species. Although the North Atlantic clade (Clade D) showed a relatively close relationship with Clade C, the genetic difference between Clades C and D was comparable to that between Clades A and B. Based on this, Clade D could be considered a different species. However, comprehensive sampling in both the northern Pacific and Arctic Oceans is necessary to determine whether they should be recognized as distinct species. Based on the results of this study, it is safe to state that “ *E. emarginata* ” is not a single species but a species complex. A comparison based on morphology and DNA sequences, covering the distribution range of this species complex, is required to resolve this taxonomic issue.

Clade A was the only clade distributed in both the Sea of Japan and the Pacific Ocean, but no COI haplotypes and ITS2 genotypes were shared between the two regions. In contrast, the northern and southern local populations in the Pacific Ocean (P‐NTS and P‐STS), which were more distant from each other, shared some haplotypes (Figure 3). Consistent with these patterns, the pairwise *F*_ST_ estimates revealed significant differences between the Sea of Japan and Pacific Ocean populations (Table 3). These results indicated that dispersal between the Sea of Japan and the Pacific Ocean is currently restricted. The deep‐sea isolation event caused by the uplifting of the Japanese Archipelago occurred ~4.5–1.7 Ma. The divergence age between Clades A and B was estimated to have occurred at ~2.72 Ma. As there were no differentiating factors before the isolation of the deep sea, it may be reasonable to attribute the differentiation of Clades A and B to the uplift of the Japanese Archipelago. In this scenario, divergence within Clade A (Sea of Japan vs. Pacific Ocean: 0.42 Ma) indicates exceptional dispersal across the strait.

Water temperature is assumed to be one of the most likely factors driving this dispersion. The distribution of “ *E. emarginata* ” has been reported at shallower depths at higher latitudes (2–2200 m) (Vassilenko 1989), indicating that water temperature determines the upper limit of its bathymetric distribution. In our study, all Pacific clades were collected from shallower depths in colder northern areas. If this were the case, the distribution of “ *E. emarginata* ” in Japanese waters would extend to shallower depths than the present during cold periods, facilitating dispersal over the shallow strait. Given that no recent dispersal has been detected, one possible explanation is that the present‐day inflow of warm currents into the Tsugaru Strait, along with its high temperatures, might limit the dispersal of this species under present‐day conditions.

The estimated divergence time indicates that migration between populations in the Sea of Japan and the Pacific Ocean has been restricted for the past 400,000 years. Given this estimation, populations in the Sea of Japan likely endured glacial anoxic events, such as those occurring during the LGM (~20,000 years ago). The local populations J‐NTS and J‐STS showed lower haplotype and nucleotide diversity and significantly negative values for both Tajima's *D* and Fu and Li's *F*_s_ (Table 4). These values have also been reported for other species in the Sea of Japan as indicators of bottlenecks due to anoxic events, followed by subsequent expansion (e.g., Fujita et al. 2021). Considering that the ancestors survived in the Sea of Japan during the last glacial period, signs of a population bottleneck are expected to persist. Notably, Fu's *F*_s_ statistics were not significant for the local populations in the Sea of Japan. Given that Fu's *F*_s_ is particularly sensitive to the emergence of novel haplotypes, the lack of significance may reflect limited post‐bottleneck diversification. This could indicate that, despite signs of demographic recovery, insufficient time has passed or that ecological constraints and small population sizes may have prevented haplotype diversification. This pattern does not conflict with the recovery of population size from an anoxic bottleneck during the LGM.

In contrast, the local population J‐TS showed no genetic signal consistent with an anoxic bottleneck. Generally, the local population within a refugium sustains high genetic diversity. A northern refugium is thought to have been established around the area of J‐TS during the last glacial period. Continuous current inflow from the southern strait (Korea Strait, ~130 m) has been suggested to have formed refugia during the last glacial period (Gorbarenko and Southon 2000; Kokita and Nohara 2011). The depth of the Tsugaru Strait (~130 m) is comparable to that of the Korea Strait, and the current inflow might have occurred during the same period. The Oyashio Current is believed to have flowed from the Tsugaru Strait to the central area of the Sea of Japan soon after the LGM (Koizumi et al. 2006). If the current inflow and/or seawater exchange through the Tsugaru Strait were maintained during the last glacial period, it would not be surprising if the region near the Tsugaru Strait was an exceptionally oxic and high‐salinity refugium. Given that only one local population was unaffected by anoxia, it is unlikely that it survived in the Sea of Japan after retreating to a specific depth. This indicates that factors other than the depth may have contributed to this finding. However, because of low sampling densities, the effect of water depth must be assessed with caution. Further studies with denser sampling in the bathymetric direction and estimation of past changes in distribution are required to determine the influence of depth.

Neutrality tests revealed contrasting demographic histories among the three local Pacific populations (Table 4). For P‐TS, we caution that the interpretation may be limited because of the small sample size. The results for P‐NTS suggest potential changes in population structure. Given the high haplotype diversity observed in P‐NTS, a recent bottleneck appears unlikely. One possible explanation could be gene flow from external populations. However, this remains speculative, as our current dataset does not allow for a conclusive assessment. Further genome‐wide analyses are required to clarify these patterns.

Molecular phylogenetic analyses focusing on cumaceans are limited (Haye et al. 2004; Rehm et al. 2020; Uhlir et al. 2021; Gerken et al. 2022). In the present study, the first phylogeographical analysis confirmed the presence of three clades of “ *E. emarginata* ” only in the Northwest Pacific. Such regional phylogenetic groups (or cryptic species) will increase with more extensive research. We hypothesized that the inflow of oxygen‐rich seawater from the Tsugaru Strait maintained suitable conditions in the area facing the strait during glacial periods. The “ *E. emarginata* ” species complex appears to reflect historical population dynamics, and may provide crucial insight into long‐term population dynamics in northern high latitudes. Future studies should implement genome‐wide strategies to investigate this hypothesis and explore long‐term population dynamics by analyzing high‐resolution genetic structure.

## Author Contributions

**Kai Okamoto:** conceptualization (equal), data curation, formal analysis, investigation (equal), methodology, visualization, writing – original draft. **Shigeaki Kojima:** conceptualization (equal), investigation (equal), project administration, funding acquisition, supervision, writing – review and editing.

## Conflicts of Interest

The authors declare no conflicts of interest.

## Supporting information


**Appendix S1:** ece372098‐sup‐0001‐AppendixS1.zip.

**Table S1:** ece372098‐sup‐0002‐TableS1.xlsx.

## Data Availability

Individual haplotype and genotype data are deposited in the DNA Data Bank of Japan (DDBJ) (accession numbers are listed in Table S1). The DNA sequences we determined in this study are also accessible in Appendix S1.

## References

[ece372098-bib-0001] Akiyama, T. 2009. “Deep‐Sea Cumacean Crustaceans (Peracarida) Collected From Pacific Coast of Northern Honshu, Japan.” National Museum of Nature and Science Monographs 39: 483–493.

[ece372098-bib-0002] Akiyama, T. 2014. “Deep‐Sea Cumacean Crustacea From the Sea of Japan Based on the Specimens Collected by R/V *Tansei‐Maru* (Cruise KT‐11‐9).” National Museum of Nature and Science Monographs 44: 157–176.

[ece372098-bib-0003] Akiyama, T. , and S. Gamô . 2012. “The Cumacean Genus *Eudorella* (Crustacea: Peracarida) From Japanese Waters, Northwest Pacific, and *E. suluensis* sp. Nov. From the Sulu Sea, Indo‐West Pacific.” Zootaxa 3319: 1–56.

[ece372098-bib-0004] Akiyama, T. , M. Shimomura , and K. Nakamura . 2008. “Collection of Deep‐Sea Small Arthropods: Gears for Collection and Processing of Samples on Deck.” [in Japanese With English Abstract.] Taxa 24: 27–32.

[ece372098-bib-0005] Amano, K. 2004. “Biogeography and the Pleistocene Extinction of Neogastropods in the Japan Sea.” Palaeogeography Palaeoclimatology Palaeoecology 202: 245–252.

[ece372098-bib-0006] Amante, C. , and B. W. Eakins . 2009. “ETOPO1 Arc‐Minute Global Relief Model: Procedures, Data Sources and Analysis.” Vol. 24. *NOAA Technical Memorandum NESDIS NGDC*.

[ece372098-bib-0007] Bishop, J. D. D. , and S. H. Shalla . 1994. “Discrete Seasonal Reproduction in an Abyssal Peracarid Crustacean.” Deep Sea Research Part 1: Oceanographic Research Papers 41: 1789–1800.

[ece372098-bib-0008] Brandt, A. , I. Alalykina , S. Brix , et al. 2019. “Depth Zonation of Northwest Pacific Deep‐Sea Macrofauna.” Progress in Oceanography 176: 102131.

[ece372098-bib-0009] Brandt, A. , M. Błażewicz‐Paszkowycz , R. N. Bamber , et al. 2012. “Are There Widespread Peracarid Species in the Deep Sea (Crustacea: Malacostraca)?” Polish Polar Research 33: 139–162.

[ece372098-bib-0010] Brenke, N. 2005. “An Epibenthic Sledge for Operations on Marine Soft Bottom and Bedrock.” Marine Technology Society Journal 39: 10–21.

[ece372098-bib-0011] Edler, D. , J. Klein , A. Antonelli , and D. Silvestro . 2021. “raxmlGUI 2.0: A Graphical Interface and Toolkit for Phylogenetic Analyses Using RAxML.” Methods in Ecology and Evolution 12: 373–377.

[ece372098-bib-0012] Excoffier, L. , and H. E. L. Lischer . 2010. “Arlequin Suite Ver 3.5: A New Series of Programs to Perform Population Genetics Analyses Under Linux and Windows.” Molecular Ecology Resources 10: 564–567. 21565059 10.1111/j.1755-0998.2010.02847.x

[ece372098-bib-0013] Folmer, O. , M. Black , W. Hoeh , R. Lutz , and R. Vrijenhoek . 1994. “DNA Primers for Amplification of Mitochondrial Cytochrome c Oxidase Subunit I From Diverse Metazoan Invertebrates.” Molecular Marine Biology and Biotechnology 3: 294–299. 7881515

[ece372098-bib-0015] Fujita, J. , D. T. Drumm , A. Iguchi , et al. 2017. “Deep‐Sea Phylogeographic Structure Shaped by Paleoenvironmental Changes and Ongoing Ocean Currents Around the Sea of Japan in a Crangonid Shrimp, *Argis lar* .” Zoological Science 34: 406–413. 28990468 10.2108/zs170014

[ece372098-bib-0014] Fujita, J. , D. T. Drumm , A. Iguchi , O. Tominaga , Y. Kai , and Y. Yamashita . 2021. “Small vs. Large Eggs: Comparative Population Connectivity and Demographic History Along a Depth Gradient in Deep‐Sea Crangonid *Argis* Shrimps.” Biological Journal of the Linnean Society 134: 650–666.

[ece372098-bib-0016] Gerken, S. , K. Meland , and H. Glenner . 2022. “First Multigene Phylogeny of Cumacea (Crustacea: Peracarida).” Zoologica Scripta 51: 460–477.

[ece372098-bib-0017] Gorbarenko, S. A. , and J. R. Southon . 2000. “Detailed Japan Sea Paleoceanography During the Last 25 kyr: Constraints From AMS Dating and δ^18^O of Planktonic Foraminifera.” Palaeogeography Palaeoclimatology Palaeoecology 156: 177–193.

[ece372098-bib-0018] Haye, P. A. , I. Kornfield , and L. Watling . 2004. “Molecular Insights Into Cumacean Family Relationships (Crustacea, Cumacea).” Molecular Phylogenetics and Evolution 30: 798–809. 15012957 10.1016/j.ympev.2003.08.003

[ece372098-bib-0019] Hewitt, G. M. 2004. “Genetic Consequences of Climatic Oscillations in the Quaternary.” Philosophical Transactions of the Royal Society of London. Series B, Biological Sciences 359: 183–195. 15101575 10.1098/rstb.2003.1388 PMC1693318

[ece372098-bib-0020] Hey, J. , Y. Chung , A. Sethuraman , et al. 2018. “Phylogeny Estimation by Integration Over Isolation With Migration Models.” Molecular Biology and Evolution 35: 2805–2818. 30137463 10.1093/molbev/msy162 PMC6231491

[ece372098-bib-0021] Hirase, S. , and M. Ikeda . 2014. “Long‐Term Vicariance and Post‐Glacial Expansion in the Japanese Rocky Intertidal Goby *Chaenogobius annularis* .” Marine Ecology Progress Series 499: 217–231.

[ece372098-bib-0022] Hirase, S. , H. Takeshima , M. Nishida , and W. Iwasaki . 2016. “Parallel Mitogenome Sequencing Alleviates Random Rooting Effect in Phylogeography.” Genome Biology and Evolution 8: 1267–1278. 27016485 10.1093/gbe/evw063 PMC4860695

[ece372098-bib-0023] Iguchi, A. , S. Takai , M. Ueno , T. Maeda , T. Minami , and I. Hayashi . 2007. “Comparative Analysis on the Genetic Population Structures of the Deep‐Sea Whelks *Buccinum tsubai* and *Neptunea constricta* in the Sea of Japan.” Marine Biology 151: 31–39.

[ece372098-bib-0024] Itaki, T. 2016. “Transitional Changes in Microfossil Assemblages in the Japan Sea From the Late Pliocene to Early Pleistocene Related to Global Climatic and Local Tectonic Events.” Progress in Earth and Planetary Science 3: 1–21.

[ece372098-bib-0025] Itaki, T. , K. Ikehara , I. Motoyama , and S. Hasegawa . 2004. “Abrupt Ventilation Changes in the Japan Sea Over the Last 30 ky: Evidence From Deep‐Dwelling Radiolarians.” Palaeogeography Palaeoclimatology Palaeoecology 208: 263–278.

[ece372098-bib-0026] Jarman, S. N. , S. Nicol , N. G. Elliott , and A. McMinn . 2000. “28S rDNA Evolution in the Eumalacostraca and the Phylogenetic Position of Krill.” Molecular Phylogenetics and Evolution 17: 26–36. 11020302 10.1006/mpev.2000.0823

[ece372098-bib-0027] Knowlton, N. , and L. A. Weigt . 1998. “New Dates and New Rates for Divergence Across the Isthmus of Panama.” Proceedings of the Royal Society of London. Series B: Biological Sciences 265: 2257–2263.

[ece372098-bib-0028] Koizumi, I. , R. Tada , H. Narita , et al. 2006. “Paleoceanographic History Around the Tsugaru Strait Between the Japan Sea and the Northwest Pacific Ocean Since 30 Cal Kyr BP.” Palaeogeography Palaeoclimatology Palaeoecology 232: 36–52.

[ece372098-bib-0029] Kojima, S. , R. Segawa , I. Hayashi , and M. Okiyama . 2001. “Phylogeography of a Deep‐Sea Demersal Fish, *Bothrocara hollandi* , in the Japan Sea.” Marine Ecology Progress Series 217: 135–143.

[ece372098-bib-0030] Kokita, T. , and K. Nohara . 2011. “Phylogeography and Historical Demography of the Anadromous Fish *Leucopsarion petersii* in Relation to Geological History and Oceanography Around the Japanese Archipelago.” Molecular Ecology 20: 143–164. 21062386 10.1111/j.1365-294X.2010.04920.x

[ece372098-bib-0031] Krøyer, H. N. 1846. “On Cumaceerne Familie.” Naturhistorisk Tidsskrift 2: 123–211. pls 1–2.

[ece372098-bib-0032] Leigh, J. W. , and D. Bryant . 2015. “PopART: Full‐Feature Software for Haplotype Network Construction.” Methods in Ecology and Evolution 6: 1110–1116.

[ece372098-bib-0033] Loeza‐Quintana, T. , C. M. Carr , T. Khan , et al. 2019. “Recalibrating the Molecular Clock for Arctic Marine Invertebrates Based on DNA Barcodes.” Genome 62: 200–216. 30461309 10.1139/gen-2018-0107

[ece372098-bib-0034] Miller, K. G. , J. V. Browning , W. J. Schmelz , R. E. Kopp , G. S. Mountain , and J. D. Wright . 2020. “Cenozoic Sea‐Level and Cryospheric Evolution From Deep‐Sea Geochemical and Continental Margin Records.” Science Advances 6: eaaz1346. 32440543 10.1126/sciadv.aaz1346 PMC7228749

[ece372098-bib-0035] Oba, T. , and T. Irino . 2012. “Sea Level at the Last Glacial Maximum, Constrained by Oxygen Isotopic Curves of Planktonic Foraminifera in the Japan Sea.” Journal of Quaternary Science 27: 941–947.

[ece372098-bib-0036] Rehm, P. , S. Thatje , F. Leese , and C. Held . 2020. “Phylogenetic Relationship Within Cumacea (Crustacea: Peracarida) and Genetic Variability of Two Antarctic Species of the Family Leuconidae.” Scientia Marina 84: 385–392.

[ece372098-bib-0037] Rozas, J. , A. Ferrer‐Mata , J. C. Sánchez‐DelBarrio , et al. 2017. “DnaSP 6: DNA Sequence Polymorphism Analysis of Large Data Sets.” Molecular Biology and Evolution 34, no. 12: 3299–3302. 29029172 10.1093/molbev/msx248

[ece372098-bib-0038] Sakuma, K. , A. Yoshikawa , T. Goto , K. Fujiwara , and Y. Ueda . 2019. “Delineating Management Units for Pacific Cod ( *Gadus macrocephalus* ) in the Sea of Japan.” Estuarine, Coastal and Shelf Science 229: 106401.

[ece372098-bib-0039] Sanders, H. L. , and J. F. Grassle . 1971. “The Interactions of Diversity, Distribution and Mode of Reproduction Among Major Groupings of Deep‐Sea Benthos.” In Proceedings of Joint Oceanographic Assembly, edited by M. Uda , 260–262. Japan Society for the Promotion of Science.

[ece372098-bib-0040] Sato, T. , N. Sato , M. Yamasaki , Y. Ogawa , and M. Kaneko . 2012. “Late Neogene to Quaternary Paleoenvironmental Changes in the Akita Area, Northeast Japan.” [in Japanese With English Abstract.] Journal of the Geological Society of Japan 118: 62–73.

[ece372098-bib-0041] Tada, R. 1994. “Paleoceanographic Evolution of the Japan Sea.” Palaeogeography Palaeoclimatology Palaeoecology 108: 487–508.

[ece372098-bib-0042] Tamura, K. , G. Stecher , and S. Kumar . 2021. “MEGA11: Molecular Evolutionary Genetics Analysis Version 11.” Molecular Biology and Evolution 38: 3022–3027. 33892491 10.1093/molbev/msab120 PMC8233496

[ece372098-bib-0043] Uhlir, C. , M. Schwentner , K. Meland , et al. 2021. “Adding Pieces to the Puzzle: Insights Into Diversity and Distribution Patterns of Cumacea (Crustacea: Peracarida) From the Deep North Atlantic to the Arctic Ocean.” PeerJ 9: e12379. 34824910 10.7717/peerj.12379 PMC8590803

[ece372098-bib-0044] Vassilenko, S. V. 1989. “Arctic Ocean Cumacea.” In The Arctic Seas: Climatology, Oceanography, Geology, and Biology, edited by Y. Herman , 431–444. Springer US.

[ece372098-bib-0045] Watling, L. , and S. Gerken . 2025. “World Cumacea Database.” https://www.marinespecies.org/cumacea.

[ece372098-bib-0046] Wessel, P. , J. F. Luis , L. A. Uieda , et al. 2019. “The Generic Mapping Tools Version 6.” Geochemistry, Geophysics, Geosystems 20: 5556–5564.

[ece372098-bib-0047] White, T. J. , T. Bruns , S. J. W. T. Lee , and J. Taylor . 1990. “Amplification and Direct Sequencing of Fungal Ribosomal RNA Genes for Phylogenetics.” PCR Protocols: A Guide to Methods and Applications 18: 315–322.

[ece372098-bib-0048] Whiting, M. F. , J. C. Carpenter , Q. D. Wheeler , and W. C. Wheeler . 1997. “The Strepsiptera Problem: Phylogeny of the Holometabolous Insect Orders Inferred From 18S and 28S Ribosomal DNA Sequences and Morphology.” Systematic Biology 46: 1–68. 11975347 10.1093/sysbio/46.1.1

